# The Role of Anticoagulation in Treating Portal Hypertension

**DOI:** 10.1007/s11901-018-0406-x

**Published:** 2018-06-18

**Authors:** Laura Turco, Filippo Schepis, Erica Villa

**Affiliations:** 1 Division of Gastroenterology, Azienda Ospedaliero-Universitaria di Modena and University of Modena and Reggio Emilia, Modena, Italy; 2 WomenInHepatology Network, Modena, Italy; 3 Department of Gastroenterology, University of Modena and Reggio Emilia, Via del Pozzo 71, 41124 Modena, Italy

**Keywords:** Cirrhosis, Anticoagulation, Fibrosis, Thrombosis, Inflammation, Heparin

## Abstract

**Purpose of Review:**

To revise experimental and clinical data supporting a less traditional role of anticoagulation for treating portal hypertension in patients with cirrhosis.

**Recent Findings:**

Portal hypertension is the main driver of complications such as ascites, variceal hemorrhage, and hepatic encephalopathy, with inflammation as a key component. The traditional view of cirrhosis as a pro-hemorrhagic condition has recently changed, prothrombotic complications being recognized as frequently as the hemorrhagic ones. Several data indicate a close relationship between inflammation, prothrombotic status, worsening of hepatic fibrosis, and portal hypertension both in animal models and in patients with chronic liver disease. These findings indicate that anticoagulation may represent a potent tool to act on fibrogenesis and therefore consequently to treat portal hypertension. All anticoagulants have good to optimal safety profiles and can be used in patients with advanced chronic liver disease with confidence.

**Summary:**

Anticoagulation has a role as a pleiotropic treatment of portal hypertension in cirrhosis.

## Introduction

Portal hypertension (PH) is the most common complication of cirrhosis and the main determinant of clinical liver decompensation, including ascites, variceal bleeding, and hepatic encephalopathy. PH initially results from increased intrahepatic resistance to blood flow. Later, it is worsened by splanchnic vasodilation (and increased blood flow to the portal vein) [1, 2••], with systemic inflammation acting as a trigger for the shift from compensated to decompensated status [2••]. Increased resistances to portal blood flow derive from both architectural alterations of the liver parenchyma and dynamic increase in the hepatic vascular tone [3, 4]. Architectural distortion of the cirrhotic liver is partly due to excessive synthesis of extracellular matrix components due to dysregulated activation of fibrogenic cells such as hepatic stellate cells (HSC) and portal myofibroblasts [5]. Several data suggest that liver fibrogenesis is greatly influenced by lobular inflammation [6] and thrombosis [7].

## Coagulative Balance in Patients with Liver Cirrhosis and Its Role in Fibrogenesis

Patients with liver cirrhosis were traditionally considered at high risk of bleeding episodes for the concurrent presence of thrombocytopenia, prolongation of prothrombin time (PT), and of activated partial thromboplastin time. Accordingly, guidelines recommended in patients undergoing invasive procedures the use of replacement therapy with platelets (PLT) and/or transfusion of fresh frozen plasma (FFP) [8, 9]. More recently, it has become quite clear that in patients with uncomplicated liver cirrhosis, bleeding episodes are the direct consequence of PH and not of a coagulative imbalance [9]. Indeed, they are mostly represented by hemorrhage due to ruptured varices or bleeding from portal hypertensive gastropathy/enteropathy while spontaneous bleeding from other sources is rare [10•]. This revised view of coagulation in patients with compensated cirrhosis shows that they are in a situation of “balanced hemostasis” as both procoagulant and anticoagulant factors are proportionately decreased [11]. When the coagulative balance is disrupted, e.g., by infection and acute kidney failure, it can hang towards either bleeding or thrombosis. One important implication of this view is that cirrhotic patients undergoing invasive procedures should receive prophylactic treatment with PLT or FFP not according to INR or platelet levels but on the basis of more comprehensive tests [12, 13, 14•]. Thromboelastography (TEG) or thromboelastometry (ROTEM), which assess the different phases of the coagulation cascade, are indeed much more informative by simultaneously assessing anticoagulant and procoagulant factors [13, 15].

Apart from bleeding risk assessment, this modified view of coagulation in cirrhosis has other implications. Several data have shown a close relationship between activation of coagulation and a more rapid progression of liver fibrosis [16–18]. Although non-novel, most appealing and informative regarding the relationship of procoagulative status and liver damage progression are the studies by Wanless et al. [7, 19], who showed frequent thrombotic occlusion of small intrahepatic veins and sinusoids in cirrhosis. This leads to intimal fibrosis and vein obstruction, with ensuing “parenchymal extinction” defined as the irreversible loss of contiguous hepatocytes and their replacement by fibrous tissue. Another hypothesis that involves thrombin generation considers functional activation of hepatic stellate cells by protease-activated receptors as the underlying mechanism leading to extracellular matrix deposition [20]. All these findings suggest that anticoagulation may interfere with liver fibrogenesis and consequently may be a potent tool for treating portal hypertension. In the following paragraphs, we will review experimental and clinical data supporting this less traditional role of anticoagulation.

## Experimental Studies

A relationship between coagulation and establishment and/or progression of fibrosis has been suggested since long [21, 22]. In a murine model of acute hepatitis virus infection, the authors were able to demonstrate the presence of microthrombi in the areas of tissue necrosis, with ensuing ischemic damage, injury by inflammatory mediators, and repair by scarring [23]. A further support to this hypothesis was given by Neubauer et al. [24] who suggested that during acute liver damage, a “clotting-like process” involving fibrinogen/fibrin deposition would create a “provisional matrix” attracting inflammatory and matrix-producing cells, then favoring proliferation of fibroblasts and collagen deposition. The evidence that a procoagulant state strongly favors progression of hepatic fibrosis also came from the studies by Anstee et al. [25] in the prothrombotic factor V Leiden mutant mice. These mice developed significantly higher hepatic fibrosis than control mice after chronic carbon tetrachloride (CCl_4_) exposure.

More recent studies focused on the role of coagulative abnormalities in experimental models of non-alcoholic steatohepatitis (NASH), the etiology, which is going to become the predominant cause of chronic liver disease (CLD) in the future years. Geys et al. [26] showed that the Adamts13 deficiency in a murine model of diet-induced liver steatosis is associated with development of hepatic microthrombosis in obese mice. Nault et al. [27] in a murine model exposed to the environmental contaminant 2,3,7,8-tetrachlorodibenzo-p-dioxin (TCDD), which triggers the progression of non-alcoholic fatty liver disease (NAFLD) to NASH, showed that activation of the coagulation cascade can drive hepatic inflammation and fibrosis independently of hepatic lipid accumulation.

As the evidence of the link between procoagulant status and development of fibrosis was strong, the evaluation of anticoagulation as a preventive or therapeutic measure was an obvious consequence (Table 1). Early in vitro experimental data had shown that heparin is able to inhibit both gene and protein expressions of type I procollagen and fibronectin [28]. In vivo long-term administration of low anticoagulant activity heparins (LAAH) was able to suppress the hepatic fibrosis induced by CCl_4_ or injection of porcine serum [29]. Several other studies, in CCl_4_ or thioacetamide murine models, using different drugs (dalteparin, enoxaparin, aspirin) confirmed these early results [25, 28–32]. Interestingly, the study from Cerini et al. [32] showed that anticoagulation was effective when given both prophylactically or therapeutically. The effect of enoxaparin was multifaceted. A significant improvement in HSC phenotype, as shown by decreased alpha smooth muscle actin (α-SMA) expression and liver desmin content, associated with a notable reduction in liver fibrosis was observed. Furthermore, enoxaparin-treated rats showed a marked and significant decrease in fibrin deposition in the liver in comparison with vehicle-treated rats, suggesting a reduction of thrombotic events within the liver. These findings were associated with a significant increase in portal blood flow reflecting a marked and significant reduction in hepatic vascular resistance.

**Table 1:** Effect of anticoagulation on liver fibrosis in experimental and clinical studies

Experimental studies				
Model	Type of anticoagulant	Timing of anticoagulant administration	Outcome(s)	Reference
Wild-type rat	Heparin	Not applicable	Reduced HSC activation and inhibited synthesis of procollagen and fibronectin	Li 2000 [28]
Rat: bile duct ligation	Unfractioned heparin, nadroparin, enoxaparin, tinzaparin	A week after fibrosis induction	Nadroparin, enoxaparin: reduced fibrosis, improved liver function Conventional heparin, tinzaparin: Increased fibrosis	Abdel-Salam 2005 [37]
Rat: CCl_4_ or porcine serum injection	Low anticoagulant activity heparins (LAAH)	Simultaneous at fibrosis induction	Reduced fibrosis	Li 2006 [29]
Rat: CCl_4_	LMWH	Simultaneous at fibrosis induction	Prevented hepatic fibrogenesis	Abe 2007 [30]
Rat: Thioacetamide	LMWH, aspirin	5 weeks after fibrosis induction	Reduced fibrosis	Assy 2007 [31]
Mouse: CCl_4_	Warfarin	Simultaneous at fibrosis induction	Reduced fibrosis	Anstee 2008 [25]
Mouse: High-fat diet	Dabigatran	Simultaneous at fibrosis induction	Reduced fibrin deposition, hepatic inflammation, and steatosis	Kopec 2014 [34]
Rat: CCl_4_	LMWH	12 weeks after fibrosis induction	Reduced hepatic vascular resistances and portal pressure	Cerini 2016 [32]
Mouse: CCl_4_	Different LMWH	12 h after fibrosis induction	Heparinase I (I-11) and combined heparinase III and II (III-II-5): reduced hepatic inflammation; reduced fibrosis. Heparinase III and I (III-I-9, III-I-5): no effect or stimulation effect on the hepatic fibrogenesis	Yan 2017 [36]
Rat: CCl_4_ or thioacetamide	Rivaroxaban	After ascites development	Reduced HSC expression and portal pressure	Vilaseca 2017 [33]
Rat: CCl_4_ oral or CCl_4_ inhalation or bile duct ligation	LMWH	Simultaneous or 1 week or 8 weeks after fibrosis induction in different rat models	Increased fibrosis	Fortea 2018 [35]
Clinical studies				
Etiology	Type of anticoagulant	Stage of fibrosis in human studies	Outcome(s)	Reference
HBV	LMWH	HBV-related liver fibrosis	Improved liver function, reduced collagen proliferation	Shi 2003 [47]
HBV	LMWH	HBV-related cirrhosis	Improved liver function, improved portal vein blood flow velocity, improved collagen levels	Huang 2007 [48]
Mixed	LMWH	Advanced cirrhosis	Reduced decompensation rate, reduced bacterial translocation and inflammation, improved survival	Villa 2012 [50]
HCV recurrence post liver transplant	Warfarin	Post liver transplant HCV-related liver fibrosis	Decreased fibrosis	Dhar 2015 [49]

*CCl*_*4*_ carbon tetrachloride, *HBV* hepatitis B virus, *HCV* hepatitis C virus, *HSC* hepatic stellate cells, *LMWH* low molecular weight heparin

More recently, rivaroxaban, a direct factor Xa inhibitor, was also shown to markedly reduce fibrin deposition and intrahepatic microthrombosis in CCl_4_- and thioacetamide-treated rats. Rivaroxaban also significantly decreased portal pressure in both models of cirrhosis without changes in portal blood flow, suggesting a reduction in intrahepatic vascular resistance [33••]. Another interesting study employing one of the new direct-acting oral anticoagulants (DOACs), dabigatran was performed in C57BL/6 J mice fed a high-fat diet [34]. Dabigatran significantly reduced hepatic fibrin deposition, hepatic inflammation, hepatocellular injury, and steatosis. Interestingly, weight gain in dabigatran-treated mice on high-fat diet was significantly lower than in control animals. The authors, on the basis of transcriptomic analysis that showed that thrombin inhibition with dabigatran significantly suppressed genes associated with lipid metabolism, indicated a primary role for thrombin as driver of NAFLD pathogenesis.

The only contrasting study on the role of anticoagulation for preventing fibrosis comes from Fortea et al. [35]. This study evaluated several different doses of enoxaparin as well as diverse protocols of administration in three different models of advanced cirrhosis in rats. The study was totally negative. Enoxaparin did not improve liver fibrosis, portal hypertension, or endothelial dysfunction. The authors hypothesized that these negative results were possibly due to the use of experimental models in a late stage of advanced cirrhosis, and therefore too advanced to be significantly influenced by therapeutic interventions. It should be underlined, however, that the experimental models used were different from those of the other studies: in this study, CCl_4_ was administered by inhalation or gavage instead than intra-peritoneally. Last but not least, recent data from Yan et al. [36•] indicate that different molecules of low molecular weight heparin (LMWH) might have different effects. These authors created a depolymerized LMWH library, using a specific or a combination of them (Hep I, II, or III). Only LMWH depolymerized by heparinase I (Hep I-11) was able to significantly decrease liver inflammation by suppressing TNF-α and IL-1β secretion, and minimizing hepatic fibrogenesis. Other LMWHs, with lower molecular weights than that obtained by heparinase I (Hep I-11) had scarce or absent antifibrogenic activity. This suggests that only drugs with larger molecular weight have relevant antifibrogenic activity. This could help interpreting the data obtained by Abdel-Salam et al. [37] in a bile duct-ligated rat model with three different LMWHs. While nadroparin and enoxaparin showed a beneficial effect, obtaining a significant decrease of fibrosis in comparison with controls, tinzaparin, which has a lower molecular weight than the other two, had, as a paradoxical effect, an increase in fibrosis. Overall, these data underline the need for further investigation of the relationship between LMWH structure and their activity as modulators of liver fibrosis [36•].

## Clinical Studies

Only few studies in humans have focused on the relationship between activation of coagulation, hepatic fibrosis, and subsequent development of portal hypertension (Table 1). Marra et al. [38] showed that thrombin could stimulate proliferation of cultured HSC and secretion of monocyte chemo-attractant protein-1 (MCP-1) through proteolytic activation of its receptor [38]. Upregulation of thrombin receptors was shown to occur in hepatitis C virus (HCV) and hepatitis B virus (HBV)—related acute or chronic liver disease—and at the same time, decreased plasma levels of antithrombin were shown to be correlated with more severe fibrosis in HCV infection [39]. As already shown in the experimental model by Anstee et al. [25], patients with HCV infection, when carrier of factor V Leiden mutation, have a fourfold risk of rapidly progressive fibrosis [40]. In human, it has been shown that patients with HCV carrying the prothrombin G20210A mutation (a pro-thrombotic condition) have a fivefold risk of liver fibrosis progression [41]. Factor V Leiden or prothrombin G20210A mutations were associated with an increased risk of clinically relevant liver fibrosis (defined as a liver stiffness > 8 kPa estimated by transient elastography) in the general population [42], corroborating the important role played by pro-thrombotic condition on liver fibrosis progression. On the opposite, patients with hemophilia and hepatitis C have a less severe hepatic disease than HCV-infected patients without hemophilia: in the study by Assy et al., [43] none of the hemophiliacs had histological evidence of advanced disease (bridging fibrosis and/or cirrhosis) as compared to controls.

After the increasing recognition of NASH as an etiologic factor for CLD, several studies have focused on the coagulative features in this condition, demonstrating that indeed, NASH is a prothrombotic condition. Wanless et al. [44] were among the first to show that in the progression from NAFLD to NASH-cirrhosis, the necrotic damage induced by lipid toxicity, leading to a direct inflammatory injury to hepatic veins, is followed by venous obstruction with secondary collapse, fibrous septation, and cirrhosis. Assy et al. [45] showed that about half of patients with NASH have genetic or acquired prothrombotic risk factors and that this is related with the severity of liver fibrosis. Tripodi et al. [46] later confirmed that the procoagulant imbalance in NASH is characterized by increased factor VIII and decreased protein C and antithrombin levels.

As in the animal models, the evaluation of the role in humans of anticoagulation for the treatment of fibrosis and hence of portal hypertension has a sound rationale. A study with short-term treatment with heparin or LMWH was performed a few years ago in patients with chronic hepatitis B [47]. Both treatments improved serum levels of (ALT), bilirubin, and reduced hyaluronic acid and type IV collagen concentrations compared with controls [47]. After treatment, the deposition of collagen in liver tissue was significantly decreased. These results indicate that anticoagulation inhibits hepatic stellate cells and it can be considered an applicable option for patients with CLD. In a later prospective randomized controlled clinical study [48], 75 patients were randomly divided into three groups: conventional treatment (*n* = 15), conventional treatment plus imported LMWH (*n* = 30), and conventional treatment plus indigenous LMWH (*n* = 30). Despite the short course of treatment (3 weeks), both LMWH treatment groups showed a distinct improvement in liver function tests and a marked decrease of propeptide of procollagen type III (PIIIP) and type IV collagen levels in comparison with conventional treatment. Most importantly, portal vein blood flow velocity markedly improved.

An interesting phase II study undergoing in the UK is evaluating the efficacy of anticoagulation with warfarin for prevention of fibrosis in liver transplant (LT) patients with recurrent HCV-infection [49]. The interim analysis showed a significant reduction of fibrosis 1 year after LT in anticoagulated patients. Although the recent availability of extremely effective and potent direct-acting antivirals against HCV, which can obtain sustained viral response (SVR) rate close to 100% and extensive regression of fibrosis, will probably make this therapeutic option obsolete, still, these results are extremely relevant as a proof of concept of the role of anticoagulation as antifibrotic therapy.

An indication of the results attainable with anticoagulation in terms of prevention of complications associated with portal hypertension comes from the randomized control trial performed by our group [50] for the prevention of portal vein thrombosis with LMWH. In this trial, we randomized 70 patients with cirrhosis, moderate liver failure (Child B7-C10), and no portal vein thrombosis (PVT) to receive prophylactic doses of enoxaparin (treatment arm) or no treatment (control arm) for 48 weeks. Patients treated with enoxaparin had a significantly lower incidence of PVT and had a significantly lower incidence of liver decompensation (mainly ascites) and of mortality than controls. While the prevention of PVT (9% in enoxa-treated vs. 28% in controls at 2 years) could be expected, much less obvious was the prevention of the other complications, namely decompensation (12% in enoxa-treated vs. 59% at 1 year) which was associated with a significantly improved overall survival (60 vs. 40%). In the anticoagulated group, both the incidence of bacterial infection and the markers of bacterial translocation and of systemic inflammation were significantly lower than in the control group. We hypothesized that anticoagulation acted by improving intestinal microcirculation, diminishing bacterial translocation, and the consequent systemic inflammation, one of the main drivers of decompensation [1, 2••]. Most relevant was the fact that the positive effect associated with enoxaparin was maintained for a prolonged period of time after stopping treatment, which is consistent with a mechanism of slow return to the baseline conditions of altered intestinal microcirculation. These observations provide a rationale to suggest that anticoagulation in patients with cirrhosis may have beneficial effects on liver function and portal hemodynamics beyond prevention of portal vein thrombosis, by counteracting the mechanisms of PH worsening, which develop outside the liver when splanchnic vasodilation takes place. The lack so far of other effective antifibrotic treatment indicates in anticoagulation a possibility ready at hand, although prospective randomized confirmatory studies would be necessary. Ongoing registered trials exploring the effect of anticoagulation on hard clinical outcomes in cirrhotic patients are reported in Table 2. Among these studies, a multicenter trial proposed in France with LMWH (Childbenox) was prematurely discontinued because of low rate of enrollment. The others are still recruiting, but no preliminary data are available.

**Table 2:** Ongoing trials on anticoagulation in patients with cirrhosis (data accessed from ClinicalTrials.gov on 6/2/2018)

Name of the study	Status	Target	Type of anticoagulation	Study info	Outcome(s)	Time frame
Effect of rivaroxaban on survival and development of complications of portal hypertension in patients with cirrhosis (CIRROXABAN)	Ongoing	Patients with advanced cirrhosis (CHILD B7-C10) and CSPH	Rivaroxaban 10 mg/daily vs placebo	NCT02643212 Multicenter prospective randomized trial	Primary: Transplant-free survival and decompensation/complications of portal hypertension Secondary: effect on liver function, portal hypertension, and systemic inflammation	2 years
Impact on morbidity and mortality of prophylactic dosing of low molecular heparin in Child-Pugh B Cirrhotic Patients (Childbenox)	Suspended	Patients with advanced cirrhosis (CHILD B7-C10)	Enoxaparin 4000 IU/daily for 2 years	NCT02271295 Interventional randomized controlled trial	Primary: Morbidity and mortality Secondary: effect on liver function, fibrosis, and portal hypertension parameters	2 years
Anticoagulation for advanced cirrhosis after TIPS	Ongoing	Patients with advanced cirrhosis (CHILD B7-C13) with TIPS	Nadroparin: 4100 IU/daily for 1 year Or Enoxaparin: 4000 IU/daily for 1 year	NCT03005444 Multicenter randomized controlled trial	Primary: Transplant-free survival Secondary: effect on liver function and on further decompensation	2 years
Safety and efficacy of bemiparin in the prevention of thrombotic events in hospitalized cirrhotic patients (BEMI-2015)	Ongoing	Patients with cirrhosis admitted to the hospital because of decompensation events for at least 3 days	Bemiparin 3.500 U/daily during hospitalization	NCT02802605 Randomized and controlled trial	Primary: Bleeding events and liver toxicity Secondary: effect on liver fibrosis and serum TGF	90 days

*CSPH* clinically significant portal hypertension, *TIPS* transjugular intrahepatic portosystemic shunt

## Safety

Safety of anticoagulation in patients with cirrhosis has always been a matter of debate. However, the change of view, which has occurred in the last years, regarding hemorrhagic risk due to altered coagulative balance of patients with cirrhosis, has also greatly modified the attitude toward anticoagulation. Among the various categories of anticoagulants, only vitamin K antagonists (VKA such as warfarin and acenocoumarol) are still considered to have a narrower therapeutic window and to carry higher risk of bleeding complications in patients with liver disease. Indeed, a retrospective analysis of 63 patients with cirrhosis and PVT treated with VKA showed an increased risk of minor bleeding episodes [51]. On the whole, however, this did not negatively influence survival, and portal hypertension, rather than anticoagulants, was found as the major driver of major bleedings in these patients [51].

For unfractioned heparin (UH) and LMWHs, there is ample evidence of their safety, either when used for treating PVT [52] or for preventing it [50]. A recent meta-analysis on the effects of anticoagulants in patients with cirrhosis and PVT showed that anticoagulated patients did not have an excess of major and minor bleedings and, most importantly, had less incidence of variceal bleeding [52]. When used prophylactically [50], enoxaparin, 4000 IU daily, was well tolerated and showed a very good safety profile. No significant difference during active period between treatment group and controls was found in terms of bleeding events (only 3 out of 70 patients experienced bleeding events from esophageal varices, 2 in the treatment arm and 1 in control arm without significant difference), or in terms of hemoglobin levels. Safety of anticoagulation has been demonstrated also in patients with cirrhosis undergoing invasive procedures [12, 53, 54•]. Anticoagulation did not impact outcome in patients with active upper gastrointestinal bleeding [53] or in those undergoing elective variceal band ligation procedures for primary or secondary prophylaxis [54•]. The bleeding risk of these patients, rather than to anticoagulation, is related to the severity of portal hypertension (larger size of esophageal varices, higher number of bands used for variceal band ligation) or to the concomitant presence of multiorgan dysfunction expressed by SOFA score and of comorbidities [10, 53, 54•].

Less information on safety in patients with cirrhosis is available on direct oral anti coagulants (DOACs) as registration studies excluded patients with liver disease, for concern about increased risk of bleeding in the absence of reversal agents. De Gottardi et al. [55] retrospectively evaluated antithrombotic treatment with DOACs in patients with splanchnic vein thrombosis and cirrhosis and concluded for a substantial effectiveness and safety of these drugs. Furthermore, for all DOACs, reversal agents are now available.

## Conclusions

The available data, both experimental and clinical, strongly support the link between altered coagulation, occurrence of microvascular thrombosis (“parenchymal extinction”) [7, 19], inflammation, and fibrotic evolution of the liver parenchyma. In patients with chronic liver disease, alterations of coagulation are usually not early detectable due to their occurrence after the establishment of cirrhosis and severe portal hypertension. Therefore, for earlier stages, etiology-targeted therapies, like direct-acting antiviral for HCV or nucleoside analogues for HBV, are the treatment of choice to prevent occurrence and progression of fibrosis and consequent onset of portal hypertension. However, for patients with more advanced disease or those with NASH, effective antifibrotic therapies are still lacking and therefore therapeutic anticoagulation could be a relevant option to be validated. A drawback so far for an extensive application of anticoagulation as antifibrotic therapy and therefore as a treatment for portal hypertension was the fact that the most evaluated category of drugs and the one with an optimal safety profile (i.e., LMWHs) required parenteral administration, thus decreasing compliance in patients often already overloaded with medicines. The availability of the new DOACs will hopefully allow overcoming this difficulty. The placebo-controlled prospective trial with rivaroxaban expected to be completed in 2019 (Table 2) will constitute an extremely relevant opportunity to confirm the potential role of anticoagulation as a therapeutic modality to decrease progression of fibrosis and to treat established portal hypertension.

## Abbreviations

ALT: Alanine aminotransferase
CCl_4_: Carbon tetrachloride
CLD: Chronic liver disease
DOACs: Direct-acting oral anticoagulants
FFP: Fresh frozen plasma
HBV: Hepatitis B virus
HCV: Hepatitis C virus
Hep: Heparinase
HSC: Hepatic stellate cells
HVPG: Hepatic venous pressure gradient
IL-1β: Interleukin-1 beta
LAAH: Low anticoagulant activity heparin
LMWH: Low molecular weight heparin
LT: Liver transplant
MCP-1: Monocyte chemo-attractant protein-1
NAFLD: Non-alcoholic fatty liver disease
NASH: Non-alcoholic steatohepatitis
PAR-1: Protease activated receptor-1
PH: Portal hypertension
PLT: Platelets
PIIIP: Propeptide of procollagen type III
PT: Prothrombin time
PVT: Portal vein thrombosis
ROTEM: Thromboelastometry
α-SMA: Alpha smooth muscle actin
SVR: Sustained viral response
TEG: Thromboelastography
TCDD: 2,3,7,8-Tetrachlorodibenzo-p-dioxin
TNF-α: Tumor necrosis factor alpha
VKA: Vitamin K antagonists
UH: Unfractioned heparin
