# Correction: The Role of Anticoagulation in Treating Portal Hypertension

**DOI:** 10.1007/s11901-018-0443-5

**Published:** 2018-11-16

**Authors:** Laura Turco, Filippo Schepis, Erica Villa

**Affiliations:** 1 Division of Gastroenterology, Azienda Ospedaliero-Universitaria di Modena and University of Modena and Reggio Emilia, Modena, Italy; 2 WomenInHepatology Network, Modena, Italy; 3 Department of Gastroenterology, University of Modena and Reggio Emilia, Via del Pozzo 71, 41124 Modena, Italy

**Correction: Current Hepatology Reports (2018) 17(3):200–208**

10.1007/s11901-018-0406-x

The article The Role of Anticoagulation in Treating Portal Hypertension, written by Laura Turco, Filippo Schepis and Erica Villa, was originally published electronically on the publisher’s internet portal (currently SpringerLink) on 18 June 2018 without open access.

With the author(s)’ decision to opt for Open Choice the copyright of the article changed on November 2018 to © The Author(s) 2018 and the article is forthwith distributed under the terms of the Creative Commons Attribution 4.0 International License (http://creativecommons.org/licenses/by/4.0/), which permits use, duplication, adaptation, distribution and reproduction in any medium or format, as long as you give appropriate credit to the original author(s) and the source, provide a link to the Creative Commons license and indicate if changes were made.

The original article has been corrected.

